# Conditional power and friends: The why and how of (un)planned, unblinded sample size recalculations in confirmatory trials

**DOI:** 10.1002/sim.9288

**Published:** 2022-01-13

**Authors:** Kevin Kunzmann, Michael J. Grayling, Kim May Lee, David S. Robertson, Kaspar Rufibach, James M. S. Wason

**Affiliations:** ^1^ MRC Biostatistics Unit University of Cambridge Cambridge UK; ^2^ Population Health Sciences Institute Newcastle University Newcastle upon Tyne UK; ^3^ Institute of Psychiatry Psychology and Neuroscience, King's College London UK; ^4^ Methods, Collaboration, and Outreach Group (MCO), Product Development Data Sciences F. Hoffmann‐La Roche Basel Switzerland

**Keywords:** adaptive design, conditional power, interim analysis, optimal design, predictive power, sample size recalculation

## Abstract

Adapting the final sample size of a trial to the evidence accruing during the trial is a natural way to address planning uncertainty. Since the sample size is usually determined by an argument based on the power of the trial, an interim analysis raises the question of how the final sample size should be determined conditional on the accrued information. To this end, we first review and compare common approaches to estimating conditional power, which is often used in heuristic sample size recalculation rules. We then discuss the connection of heuristic sample size recalculation and optimal two‐stage designs, demonstrating that the latter is the superior approach in a fully preplanned setting. Hence, unplanned design adaptations should only be conducted as reaction to trial‐external new evidence, operational needs to violate the originally chosen design, or post hoc changes in the optimality criterion but not as a reaction to trial‐internal data. We are able to show that commonly discussed sample size recalculation rules lead to paradoxical adaptations where an initially planned optimal design is not invariant under the adaptation rule even if the planning assumptions do not change. Finally, we propose two alternative ways of reacting to newly emerging trial‐external evidence in ways that are consistent with the originally planned design to avoid such inconsistencies.

## 1 INTRODUCTION

The planning phase of confirmatory clinical trials is typically characterized by substantial uncertainty about the magnitude of the parameters underlying the hypothesis of interest. Clinical data collection is expensive and time consuming leads to a strong economic incentive to reach the study goals with as little data as possible. The conventional statistical criteria to determine the sample size of a trial are a one‐sided type I error rate of 2.5% and a power of 80% or 90%. Since power is a function of the unknown effect size, the initial design must be specified under substantial uncertainty. Mainly, two ways of addressing this challenge have been put forward in the literature.

First, the so‐called “hybrid” approach to sample size derivation takes a Bayesian view on determining the initial sample size of a clinical trial,^1^ which requires the specification of an informative prior on the parameters of interest. This then allows reasoning about the “expected power” of a trial as a function of its sample size and, consequently, determination of the sample size such that the expected power exceeds a target threshold. This concept is a straight‐forward extension of the usual practice of computing the power under a fixed point alternative, which can be recovered as a special case when considering a point prior. The advantage lies in the fact that the a priori information about the magnitude of the effect size is faithfully reflected in the sample size derivation. Since the actual analysis is still frequentist, type I error rate control is not compromised.

Second, the authors have proposed to apply the concept of adaptive design changes to recalculate the initial sample size of a design based on data observed within the trial itself.^2^ The rationale behind this approach is to use the accruing evidence about the unknown parameters driving the sample size derivation to “correct” the sample size mid‐trial. During the interim analysis, the accrued data can either be unblinded or not. Blinded interim analysis is particularly useful if relevant nuisance parameters such as the variance are also unknown.^2^, ^3^ We focus on the unblinded case which allows for a more precise interim assessment of the effect size than methods that retain the blind. Data‐driven interim analyses introduce multiplicity issues and potentially inflate the type I error rate. The maximal type I error rate constraint can be protected by applying the conditional error principle,^4^, ^5^ an equivalent formulation via α‐spending, or combination functions.^2^ Often, the sample size of the current trial is adjusted such that the conditional power given the data observed up to the interim analysis again exceeds the initial threshold for unconditional power.^6^ Here, conditional power is the probability to reject the null hypothesis given the interim data as a function of the unknown parameters. Any recalculation based on conditional power arguments must address the problem that conditional power, just as unconditional power, depends on the unknown underlying parameters and must thus be estimated to inform a sample size recalculation. Yet, precise estimation of the relevant parameters before the conclusion of a trial is hard since only a fraction of the final sample size is available. Three approaches to estimating conditional power have been discussed in the literature.

First, in a slight abuse of terminology, the same term is often used to refer to the “assumed conditional power” that is obtained by plugging in the point alternative used for the initial sample size derivation. Evidently, this assumed conditional power makes no use of the accrued trial data since the effect size is kept fixed. Second, the authors put forward “observed conditional power,” which replaces the unknown parameters with their maximum‐likelihood estimates. The latter approach is often criticized for “…[using] the interim estimate of the effect […] twice …” Bauer et al^2^ ^(p330)^ and Bauer and König.^7^ Third, conditional power can be evaluated as Bayesian expected power conditional on the observed interim data, that is, by averaging conditional power as a function of the unknown parameters with respect to the posterior density after conducting the interim analysis. Within the hybrid Bayesian framework this is usually referred to as “predictive power”.^1^, ^2^

The idea of adjusting a sample size mid‐trial to correct potentially erroneous planning assumptions about the effect size is appealing. Conceptually, however, it is difficult to justify why methods originally derived for *unplanned* design changes should employed to this end—after all, all potential interim outcomes can be anticipated during the planning of a two‐stage trial and optimal decision rules could be preplanned.

The purpose of this article is to argue that preplanned sample size adaptations in the above sense are inefficient and unnecessary if the original design was planned optimally. However, we discuss situations where an unplanned design adaptation might still be warranted as reaction to emerging trial‐external evidence and give guidance on how such an adaptation can be implemented. We hope that this principled view on the justification of adaptive sample size recalculations aids practitioners in deciding whether an adaptation is sensible in the first place and, if justified, how to conduct it.

To this end, we first review assumed conditional‐, observed conditional‐, and predictive power from an estimation perspective. We then discuss the risks of constructing an adaptive two‐stage design by naïvely applying a conditional‐power‐based sample size recalculation rule. The drawbacks of this naïve approach directly lead to the concept of optimal‐two stage designs^8^, ^9^, ^10^ (w.l.o.g. we focus on minimal expected sample size as the optimality criterion). By definition, these optimal two‐stage designs cannot be made more efficient by sample size recalculation. However, their optimality depends on the initial trial‐external evidence that feeds into the planning assumptions. This trial‐external evidence might change during an ongoing study and may thus still mandate a sample size recalculation.

In Section 3, we then discuss, how such a recalculation interacts with the concept of optimal two‐stage designs. In particular, we demonstrate that commonly used methods are inconsistent in that they imply that that the original design needs to be modified for every possible interim outcome even if there is no change to the planning assumptions. We propose two novel methods that overcome this inconsistency.

In the following, we assume that the interest lies in testing a new treatment for efficacy. For the sake of simplicity, we consider the case of a single‐arm trial. All considerations can easily be extended to the two‐arm case (see the application example in Section 3.3). We further assume that the individually observed outcomes of the study participants $X_{i},i=1,\ldots ,n$ are iid and that their distribution has finite first moment θ and unit variance. Again, all considerations can be extended to the case of generic known variances and, at least approximately, to the case of unknown variance. A suitable test statistic for the null hypothesis of interest ${ℋ}_{0}:\theta \leq 0$ is

(1) $$Z_{n}=\frac{1}{\sqrt{n}}\overset{n}{\underset{i=1}{\sum }}X_{i}.$$

Invoking the central limit theorem, $Z_{n}\overset{.}{˜}𝒩(\sqrt{n}\;\theta ,1)$ and, on the boundary of the null hypothesis, $Z_{n}\overset{.}{˜}𝒩(0,1)$. Further assuming a maximal permissible type I error rate of α=0.025 (which we use for the remainder of the article), the critical value for a single‐stage fixed‐sample‐size design is the 1−α quantile of the standard normal distribution, that is, approximately c=1.96. The test then rejects ${ℋ}_{0}$ if and only if $Z_{n}>c$ after the outcomes of *n* subjects have been observed. The required size of the trial *n*, given a maximal allowable type I error rate, is usually determined by some form of restriction on the (minimal) statistical power of the test. Approaches to defining such a power constraint under different assumptions were reviewed in Kunzmann et al.^11^

## 2 A CRITICAL REVIEW OF PREVIOUS WORK

### 2.1 Monitoring power

After observing *m*, 0<m<n, outcomes, an independent data monitoring committee might be interested to learn about the prospects of eventually rejecting the null hypothesis. Since $Z_{n}$ and $Z_{m}$ are (asymptotically) jointly normal, the conditional distribution of $Z_{n}$ given $Z_{m}$ and θ is again normal and given by

(2) $${ℒ}_{\theta }[Z_{n}|Z_{m}=z_{m}]\overset{.}{=}𝒩\left(\sqrt{n}\;\theta +\sqrt{\tau }\left(z_{m}-\sqrt{m}\;\theta \right),\;1-\tau \right),$$

where τ:=m/n is the “information fraction” at the interim analysis (and the squared correlation between $Z_{n}$ and $Z_{m}$). The probability of rejecting the null hypothesis at the end of the trial given the interim data $Z_{m}=z_{m}$ as a function of θ is referred to as the conditional power in the literature. In the setting at hand, it is defined as

(3) $$\begin{array}{ll} \text{CP}(z_{m},c,\theta ) & :={\text{Pr}}_{\theta }[Z_{n}>c\;|\;Z_{m}=z_{m}] \end{array}$$

(4) $$\begin{array}{ll} & =1-\Phi \left(\frac{c-\sqrt{n}\;\theta -\sqrt{\tau }\;z_{m}+\sqrt{\tau }\sqrt{m}\;\theta }{\sqrt{1-\tau }}\right) \end{array}$$

(5) $$\begin{array}{ll} & =1-\Phi \left(\frac{c/\sqrt{m}+\sqrt{\tau }\;\left(\theta -{\hat{\theta }}_{m}\right)-\theta /\sqrt{\tau }}{\sqrt{\left(n-m)/(n\;m\right)}}\right). \end{array}$$

Here ${\hat{\theta }}_{m}:=1/m\;\sum_{i=1}^{m}X_{i}$ is the observed effect after *m* individuals' responses were observed. Since θ is unknown, so is $\text{CP}(z_{m},c,\theta )$ and it cannot be evaluated directly upon observing $Z_{m}=z_{m}$. Yet, as a function of the unknown quantity θ, $\text{CP}(z_{m},c,\theta )$ can be estimated from observed data. The estimation perspective on “evaluating” conditional power is less commonly taken in the literature but provides a consistent framework to compare the characteristics of different methods.^7^

First, conditional power can be estimated based on a fixed point alternative $\theta_{1}>0$. In a slight abuse of terminology, this quantity is often also referred to as “conditional power.” To clearly distinguish it from $\text{CP}(z_{m},c,\theta )$ we denote it “assumed conditional power”

(6) $$\text{ACP}(z_{m},c):=\text{CP}(\;z_{m},c,\theta_{1}\;).$$

Second, the observed effect ${\hat{\theta }}_{m}$ can be used as a plug‐in estimator for the unknown effect size.^6^ This quantity is sometimes referred to as “observed conditional power” in the literature and is defined as

(7) $$\text{OCP}(z_{m},c):=\text{CP}(z_{m},c,{\hat{\theta }}_{m}).$$

Third, a Bayesian approach can be taken if one is willing to quantify the uncertainty about the unknown parameter θ by modeling it as the realization of a random variable Θ∼φ(·) where φ(θ) is the prior probability density function evaluated at the parameter value θ. Our definition of this so‐called “predictive power”

(8) $$\begin{array}{ll} {\text{PP}}_{\varphi }(z_{m},c) & :={\mathrm{Pr}}_{\varphi }[Z_{n}>c\;|\;\Theta >0,Z_{m}=z_{m}] \end{array}$$

(9) $$\begin{array}{ll} & =\int_{0}^{\infty }\text{CP}(z_{m},c,\theta )\;\varphi (\theta \;|\;Z_{m}=z_{m},\Theta >0)d\theta \end{array}$$

differs slightly from the one proposed by Spiegelhalter et al^1^ in that we condition on a positive effect size Θ>0. This is more consistent with the notion of expected power as discussed in Kunzmann et al^11^

(10) $$\begin{array}{ll} {\text{EP}}_{\varphi }(n,c) & :={\text{Pr}}_{\varphi }[Z_{n}>c\;|\;\Theta >0] \end{array}$$

(11) $$\begin{array}{ll} & =\int {\text{PP}}_{\varphi }(z_{m},c)\;f_{\varphi }(z_{m}|\Theta >0)\;dz_{m}, \end{array}$$

where $f_{\varphi }(z_{m}|\Theta >0)$ is the probability density function of the predictive distribution of $Z_{m}|\Theta >0$. The difference is only of practical relevance when a substantial fraction of the a priori probability mass is concentrated on the null hypothesis.

**FIGURE 1:**
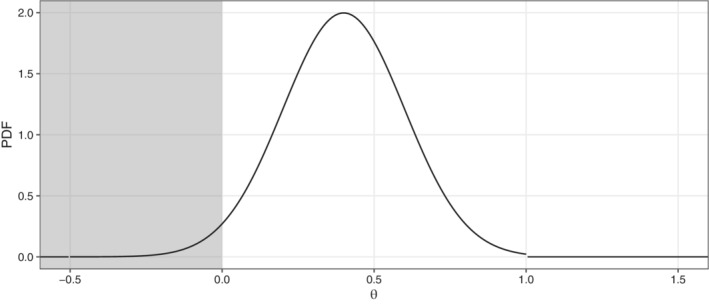
Assumed prior density function. The gray area indicates the null hypothesis, ${ℋ}_{0}$, of no effect

We now compare ACP,OCP, and PP by means of a concrete example. To this end, we assume that the available prior information can be summarized in a truncated normal prior with density

(12) $$\varphi (\theta ):=1_{\left[-0.5,1\right]}(\theta )\;\frac{\phi \left(\frac{\theta -0.4}{0.2}\right)}{\Phi \left(\frac{1-0.4}{0.2}\right)-\Phi \left(\frac{-0.5-0.4}{0.2}\right)},$$

(see Figure 1). A truncated normal prior allows the analytic computation of the posterior distribution under a normal likelihood. It is also the maximum entropy distribution on a compact interval given mean and standard deviation and thus “least informative” given a lower and upper boundary on plausible effects as well as the location and vagueness (variance) of the prior. The choice of the prior is a key consideration, but an extensive discussion is beyond the scope of this article. We refer the reader to discussion in Spiegelhalter et al,^12^ Rufibach et al,^13^ and Hampson et al,^14^ as well as the formal prior elicitation framework SHELF,^15^, ^16^ which is routinely used in practice by pharmaceutical companies.^17^

Following Reference 11, the required sample size n=79 is determined by requiring a minimal expected power of 1−β=80%. Since no point alternative is used to derive *n*, we use the prior mean rounded to the first decimal point to evaluate ACP and define $\theta_{1}:=0.4$.

**FIGURE 2:**
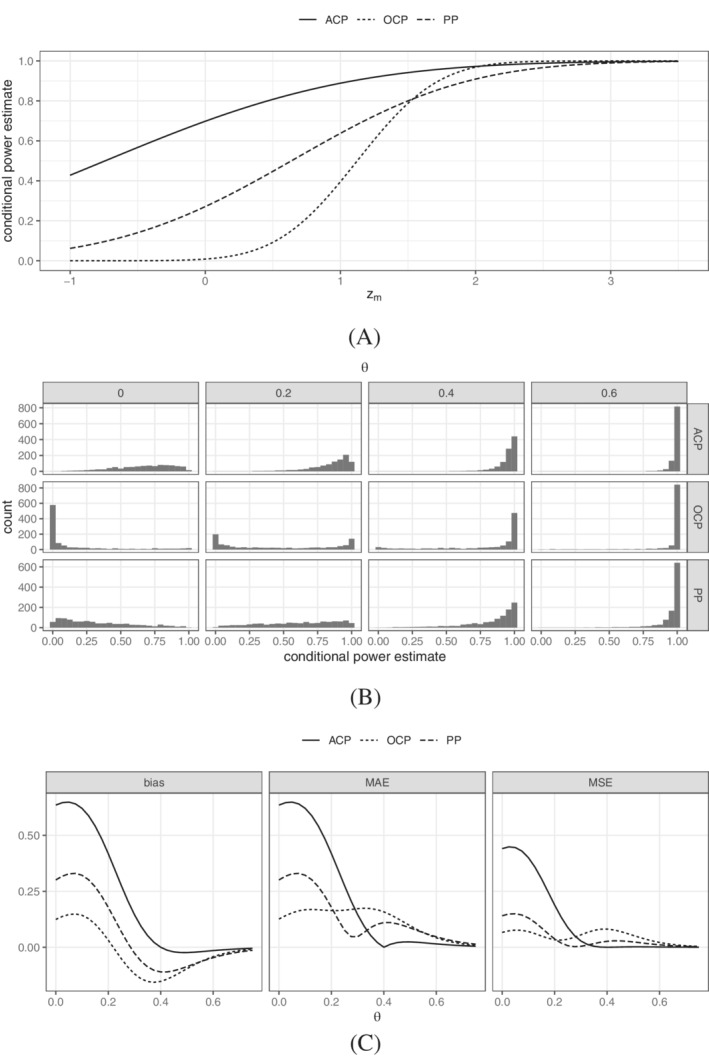
Properties of ACP,OCP, and PP as estimators of the unknown conditional power at m=26 and overall sample size n=79. (A) Estimates as function of the interim data. (B) Histograms of the sampling distributions. (C) Bias, mean absolute error (MAE), and mean squared error (MSE)

The three estimators are depicted in Figure 2A as functions of the observed interim outcome, where the interim time‐point m=26 (chosen heuristically as approximately 1/3 of the overall sample size). The most sensitive measure is OCP while ACP is the least sensitive (see also spread of the sampling distributions in Figure 2B). This is due to ACP assuming a fixed effect size and thus only being affected by direct changes in $z_{m}$. Both OCP and PP, however, are also indirectly affected by updating the belief about the effect size with the interim results. The plug‐in estimate OCP assumes that the observed effect is the true effect and does not quantify the uncertainty around this value in any way. PP, on the other hand, invokes Bayes' theorem and then integrates $\text{CP}(z_{m},c,\theta )$ with respect to the obtained posterior distribution. The degree of adaptation of PP thus depends on the vagueness of the chosen prior. In fact, as the prior approaches a point mass at $\theta_{1}$, PP converges to ACP. ACP can thus be seen as a special case of PP with a point prior on $\theta_{1}>0$.

A fundamental difference between ACP and PP on the one hand and OCP on the other hand is that OCP is the only estimator that does not condition on Θ. In contrast, ACP implicitly conditions on $\Theta =\theta_{1}>0$ and PP on Θ>0. Since OCP does not condition on Θ>0, the sampling distribution is much more left‐skewed for small effects than for the other two estimators (see Figure 2B). Also, the negligence of the sampling variation of ${\hat{\theta }}_{m}$, which OCP plugs in the expression for conditional power, leads to a larger variance for intermediate values of θ and the characteristic U‐shape.^7^ This high variance of OCP directly translates to a relatively large mean absolute error (MAE) and mean squared error (MSE) when estimating conditional power, see Figure 2C. PP is the posterior expectation of conditional power with respect to the chosen prior (conditional on a positive effect) and thus minimizes the quadratic Bayes risk, that is, the average MSE weighted by φ(·|Θ>0). This leads to relative good precision for parameter values with high a priori likelihood (around θ=0.4). If one wanted to minimize the MAE directly, the posterior median would be optimal. The principle, however, remains the same and the posterior mean is more consistent with the derivation of the initial sample size using expected power. ACP is clearly the best in terms of bias and MAE/MSE for values close to or above $\theta_{1}$, but its performance as an estimator of the conditional power quickly deteriorates for small effect sizes.

PP is thus the natural choice for monitoring power during the course of a trial since it strikes a prior‐evidence‐driven compromise between the properties of ACP (low prior variance) and OCP (high prior variance).

### 2.2 Naïve unplanned sample size adaptations

Monitoring the predictive power of an ongoing trial naturally raises the question of whether this information can be leveraged to improve the operating characteristics of a trial. Typically, sample size recalculation is considered in the context of group‐sequential designs at the prespecified interim analysis.^2^, ^6^, ^8^ Yet, for the sake of simplicity, the following considerations are based on a single‐stage design with fixed *n* and *c*. We restrict the discussion to a single unplanned interim analysis. All results can be generalized to the case of more complex starting designs and multiple interim analyses.

A method for sample size recalculation often discussed in the literature is to derive a new sample size $n^{\prime }$ and a new critical value $c^{\prime }$ such that some estimate of conditional power exceeds a threshold $1-\tilde{\beta }(z_{m})$. This threshold may or may not vary with the observed interim result. Different choices for the estimator of conditional power and the choice of $\tilde{\beta }(z_{m})$ have been discussed in the literature.^2^, ^6^, ^8^ Strict overall type I error rate control can be maintained by invoking the conditional error principle,^4^, ^5^, ^18^ that is, by limiting the maximal conditional type I error rate of the new design to the maximal conditional type I error rate of the original design. Often, trial protocols leave the exact choice of $1-\tilde{\beta }(z_{m})$ open since it can be chosen ad hoc without compromising strict type I‐error rate control. The basic concept of adjusting the sample size to achieve the desired conditional power is, however, a common approach, see, for instance, Bhatt et al^19^ and Mehta et al.^20^

To be consistent with the derivation of the initial sample size of n=79 in the example considered earlier, we use predictive power as an estimator of conditional power. Furthermore, we set $\tilde{\beta }(z_{m})=\beta =0.2$. We indicate the dependency on the final sample size and the interim time point explicitly by redefining

(13) $$\begin{array}{ll} \text{CP}(m,n,z_{m},c,\theta ) & :={\text{Pr}}_{\theta }[Z_{n}>c\;|\;Z_{m}=z_{m}], \end{array}$$

(14) $$\begin{array}{ll} {\text{PP}}_{\varphi }(m,n,z_{m},c): & ={\text{Pr}}_{\varphi }[Z_{n}>c\;|\;\Theta >0,Z_{m}=z_{m}]\;. \end{array}$$

For given $Z_{m}=z_{m}$, the recalculation rule then corresponds to solving the optimization problem

(15) $$\begin{array}{ll} \underset{n^{\prime },c^{\prime }}{\text{argmin:}}\; & n^{\prime }, \end{array}$$

(16) $$\begin{array}{ll} \text{subject to:}\; & \text{CP}(m,n^{\prime },z_{m},c^{\prime },0)\leq \;{\text{CP}}_{n}(m,n,z_{m},c,0), \end{array}$$

(17) $$\begin{array}{ll} & {\text{PP}}_{\varphi }(m,n^{\prime },z_{m},c^{\prime })\geq \;1-\tilde{\beta }(z_{m}), \end{array}$$

(18) $$\begin{array}{ll} & n^{\prime }\geq \;n_{\min}>m, \end{array}$$

(19) $$\begin{array}{ll} & n^{\prime }\leq \;n_{\max}. \end{array}$$

Constraint (16) implements the conditional error principle by limiting the conditional (type I) error rate under the new design to the conditional (type I) error rate under the original design. The minimal sample size constraint (18) allows $n_{\min}>m$ for cases where a minimal sample size upon rejection of the null hypothesis is deemed necessary. Note that the trial cannot stop at $n^{\prime }=m$ without immediately accepting the null hypothesis since the conditional error under the new design in the case of early acceptance would be 1 but is always smaller than 1 for the original single‐stage design. Imposing a maximal sample size of $n_{\max}$ via constraint (19) is a practical necessity since the recalculated sample size could otherwise tend to infinity as $z_{m}$ approaches negative infinity. In cases where this constraint prevents a solution (low observed effect), the trial is usually stopped early for futility declaring that the null hypothesis cannot be rejected.

**FIGURE 3:**
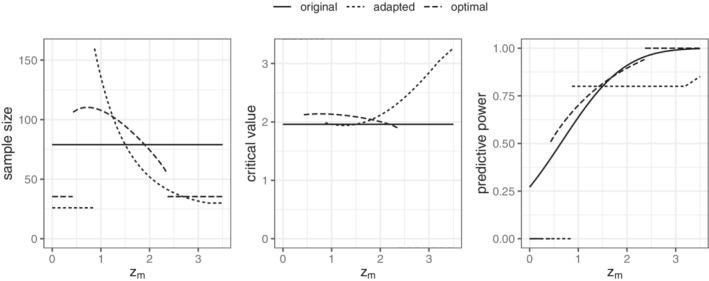
Original single‐stage design; naïvely adapted design with sample size $n^{\prime }(z_{m})$ and critical value $c^{\prime }(z_{m})$ functions defined by the conditional optimization problem (15) to (19) and $n=79,m=26,n_{\max}=160,n_{\min}=30$, and $1-\tilde{\beta }(z_{m})=0.8$; the optimal design is the solution of (20) to (22)

If the recalculation rule defined in (15) to (19) was made mandatory in the study protocol, it yields two functions $n(z_{m})$ and $c(z_{m})$ as the point‐wise solution of the problem which jointly define an adaptive design. Here, “mandatory” means that it is decided a priori to always (for all $z_{m}$) recalculate the final sample size and critical value in the above specified way after a fixed number of outcomes *m* has been observed. The term “adaptive design” is slightly misleading in this context. The design itself is prespecified (and thus not adapted or changed) but only *n* and *c* are adaptive since they vary as functions of $z_{m}$. The corresponding sample size and critical value functions for m=26 are depicted in Figure 3.

The mandatory application of the adaptation rule results in a two‐stage design and the final sample size is thus a random variable $n(Z_{m})$. Optimality can thus no longer be defined in terms of the (random) sample size itself but only in terms of a functional of the distribution of $n(Z_{m})$. For instance, one could use a weighted sum, $E[n(Z_{m})]+\eta \sqrt{V[n(Z_{m})]}$, of the expected value and standard deviation of the sample size as the objective criterion. The rationale for adding a penalty depending on the standard deviation of the sample size is that it might incur additional costs due to more complicated logistics (eg, on demand production of additional drug doses). For the single‐stage design, this proposal always reduces to the fixed sample size *n*. Whether or not the two‐stage design is considered better then depends on the choice of η (the relative weight of the standard deviation in the objective). In the particular situation considered here, the expected sample size is 48.3 and its standard deviation is 30.1. Since the original *n* was 79, the two‐stage design would be considered better than the single‐stage design for η<1.02.

This comparison ignores the fact that the original unconditional constraint of an expected power of more than 80% is no longer fulfilled by the design with mandatory sample size adaptation. Both expected power (66.9% vs 80.0%) and maximal type I error rate (1.8% vs 2.5%) are lower than for the original design. The difference in operating characteristics is mainly due to the fact that the new design implicitly introduces a binding early‐stopping boundary for futility when the recalculation problem does not yield a $n^{\prime }\leq n_{\max}$. Similar to suggestions in the literature on binding futility stopping, one could modify the initial nominal α and the conditional power threshold $\tilde{\beta }(z_{m})$ until the recalculated design again fulfills the required operating characteristics.^8^ This approach to defining a fully prespecified two‐stage design is, however, unnecessarily complicated in that it uses methods which are originally intended for *unplanned* design adaptations and that they still implicitly depend on a design that is never actually realized via the conditional error constraint (16).

### 2.3 Optimal preplanned sample size adaptations

Instead, the interim time‐point *m*, the sample size function $n(z_{m})$, the critical value function $c(z_{m})$, and the early futility‐ and efficacy boundaries ($n(z_{m})=m$ and $c(z_{m})=\pm \infty$) can be optimized directly. For the sample size function, Brannath and Bauer^8^ discussed this approach by fitting a polynomial of degree 4 but they did not optimize any of the other parameters. Pilz et al^9^ approached the problem in a more general form using variational methods. An R implementation that uses cubic splines for both the *n* and *c* functions is available via the package adoptr.^10^

In the following, we restrict the considerations to the case where only expected sample size is of interest and the variability of the sample size is ignored, that is, η=0. The corresponding optimal two‐stage design is the solution of

(20) $$\begin{array}{ll} {\text{argmin}}_{m,n(\cdot ),c(\cdot )}\; & E_{\varphi }[n(Z_{m})], \end{array}$$

(21) $$\begin{array}{ll} \text{subject to:}\; & {\text{Pr}}_{0}[Z_{n(Z_{m})}>c(Z_{m})]\leq \alpha , \end{array}$$

(22) $$\begin{array}{ll} & {\text{EP}}_{\varphi }(n(\cdot ),c(\cdot ))\geq 1-\beta \end{array}$$

and can be derived numerically using the R package adoptr.^10^ In the following, “optimal” always refers to optimal with respect to expected sample size in the above sense. Here, $E_{\varphi }[n(Z_{m})]$ is the expected sample size under the prior φ and expected power needs to be redefined to account for the fact that *n* and *c* are now functions of the interim results

(23) $${\text{EP}}_{\varphi }(n(\cdot ),c(\cdot )):={\text{Pr}}_{\varphi }[Z_{n(Z_{m})}>c(Z_{m})\;|\;\Theta >0].$$

Figure 3 shows the sample size‐, critical value‐, and the predictive power function in the situation discussed earlier together with the single‐stage design and the naïve adaptation based on predictive power discussed in Section 2.2. The optimal two‐stage design complies with both the predictive power and the maximal type I error rate constraints and is thus comparable with the original single‐stage design in terms of sample size. The expected sample size is much lower (56.4 vs 79) at the cost of a nonzero standard deviation of the expected sample size (28.5 vs 0) and an increased maximal sample size of 110.3. Since it is entirely prespecified, the optimal two‐stage design does not need to fall back to the conditional error principle to control the maximal type I error rate. Instead, it achieves the desired design characteristics in an optimal way since they are incorporated as constraints to problem (20) to (22). The optimal design fully exhausts the allowable maximal type I error rate and complies with the expected power constraint. This comes at the cost of a predictive power that can drop as low as 40% close to the futility boundary as shown in the right panel of Figure 3. The sample size function of the optimal design is also characteristically different from the naïve design. The latter is convex on the continuation region whereas the optimal shape has a mode close to the early‐futility boundary. A recalculation based on exceeding a fixed lower threshold for some estimator of conditional power always results in a convex sample size function and can thus never be fully optimal irrespective of how the nominal values of α and $\tilde{\beta }(z_{m})$ are chosen. For a more detailed discussion of this phenomenon, see, for example, References 8 and 9.

The fact that the optimal two‐stage design exhibits a monotonically increasing predictive power rather than a constant predictive power indicates that any recalculation rule keeping predictive power close to a fixed target value must be inefficient in terms of minimizing expected sample size. The mandatory application of the heuristic recalculation rule introduced in Section 2.2 would change the optimal design for almost every value of $z_{m}$ and thus increase expected sample size of the resulting design. This is inconsistent with the original optimization objective—by definition, no optimal design ever needs to be changed as a reaction to trial internal data alone. The direct optimization of all design parameters is superior to the mandatory application of methods for unplanned design adaptations during the planning phase of a trial. However, there are still situations in which an unplanned sample size recalculation is warranted. The optimality of a design typically depends on the chosen prior density φ and the objective function. At any point in time, the prior density encodes all trial‐external evidence about the effect size. This might change over the course of a trial. For instance, another study might publish new results before the trial is concluded and these new results might trigger a reassessment of the considerations leading to the choice of φ.

## 3 CONSISTENT UNPLANNED SAMPLE SIZE ADAPTATIONS

In the following, we discuss two approaches for reacting to trial‐external events during an unplanned interim analysis in a consistent way. Here, “consistent” means that the original design is invariant under the sample size recalculation rule for all potential interim results under the original design unless the planning assumptions or the time‐point of the interim analysis are changed. An example of an inconsistent sample size recalculation rule was given in Section 2.2: the design optimizing expected sample size is not invariant under a rule that adapts the sample size and the critical value to match a predictive power of exactly 80% (the same holds for a simpler group‐sequential design minimizing expected sample size since the predictive power of a group‐sequential trial cannot be constant on the continuation area). The following considerations are restricted to cases where the result of an adaptation is a final sample size and no further interim analyses are planned for the remainder of the trial. This means that the unplanned adaptation replaces the preplanned interim analysis although it might occur at a different point in time.

We still consider minimal expected sample size as objective criterion. Since the sample size after the interim analysis is fixed, the corresponding conditional objective criterion is the minimization of the second‐stage sample size. As in Section 2.2, the overall type I error rate is controlled via a constraint on the conditional type I error rate. W.l.o.g., we only consider cases where the original design would not stop early for futility or efficacy. Let ψ be the probability density function of the revised prior. We propose to recalculate the new sample size $n^{\prime }$ and the new critical value $c^{\prime }$ as the solution of the point‐wise (conditional on $Z_{m^{\prime }}=z_{m^{\prime }}$) optimization problem

(24) $$\begin{array}{ll} \underset{n^{\prime },c^{\prime }}{\text{argmin}}\; & n^{\prime }, \end{array}$$

(25) $$\begin{array}{ll} \text{subject to:}\; & \text{CP}(m,n^{\prime },z_{m},c^{\prime },0)\leq \text{CP}(m,n(z_{m}),z_{m},c(z_{m}),0), \end{array}$$

(26) $$\begin{array}{ll} & {\text{PP}}_{\psi }(m,n^{\prime },z_{m},c^{\prime })\geq 1-\tilde{\beta }(z_{m}). \end{array}$$

Since CP is monotonic in $n^{\prime }$, the solution is uniquely defined by the constraints, if it exists. The optimization thus reduces to finding the root of

(27) $$\begin{array}{ll} \underset{n^{\prime },c^{\prime }}{\mathrm{solve}}\; & \text{CP}(m,n^{\prime },z_{m},c^{\prime },0)=\text{CP}(m,n(z_{m}),z_{m},c(z_{m}),0), \end{array}$$

(28) $$\begin{array}{ll} & {\text{PP}}_{\psi }(m,n^{\prime },z_{m},c^{\prime })=1-\tilde{\beta }(z_{m}). \end{array}$$

The central problem is translating the unconditional (expected) power constraint of the original design problem into a conditional one for predictive power—that is, to pick a value for $\tilde{\beta }(z_{m})$. Ideally, the choice of rule for $\tilde{\beta }(z_{m})$ is such that the recalculated sample size and critical value are the same as under the original design if neither the interim timing nor the planning assumptions change. As discussed in Section 2.2, a constant value for $\tilde{\beta }(z_{m})$ does not, in general, lead to a consistent recalculation rule. Below, we propose two consistent recalculation rules, both are based on an application of the conditional error principle to the (expected) type II error rate. The first rule does not require knowledge of the underlying optimization problem for the original design. This approach is easy to implement but does not maintain the unconditional power properties of the design, which is addressed by the second proposal.

### 3.1 “Not‐worse” approach

A sensible heuristic criterion to choose $\tilde{\beta }(z_{m})$ is to require that the modified design under the new prior ψ should have at least as much predictive power as the original design under the original prior φ, that is, $\tilde{\beta }(z_{m})=1-{\text{PP}}_{\varphi }(m,n(z_{m}),z_{m},c(z_{m}))$. Consequently, we term this choice of $\tilde{\beta }(z_{m})$ the “not‐worse” approach. It follows that, for ψ=φ, the original design's $n(z_{m})$ and $c(z_{m})$ are a root of (27) and (28) and hence $n^{\prime }=n(z_{m})$, $c^{\prime }=c(z_{m})$. The original optimal design is thus indeed invariant under the proposed recalculation rule if neither the planning prior nor the interim time‐point are changed. However, the expected power of the procedure resulting from always switching to a new prior ψ≠φ will be different from the originally targeted 1−β since $1-\tilde{\beta }(z_{m})$ does not integrate to 1−β under ψ≠φ. To see this, let $f_{\psi }(z_{m}\;|\;\Theta >0)$ be the probability density function of the interim result $Z_{m}$ under θ∼ψ conditional on Θ>0, then

(29) $$\begin{array}{ll} {\text{EP}}_{\psi }(n^{\prime }(\cdot ),c^{\prime }(\cdot )) & ={\text{Pr}}_{\varphi }[Z_{n^{\prime }(Z_{m})}>c^{\prime }(Z_{m})\;|\;\Theta >0] \end{array}$$

(30) $$\begin{array}{ll} & =\int {\text{PP}}_{\psi }(m,n^{\prime }(z_{m}),z_{m},c^{\prime }(z_{m}))\;f_{\psi }(z_{m}\;|\;\Theta >0)dz_{m} \end{array}$$

(31) $$\begin{array}{ll} & =\int \underset{{\text{PP}}_{\varphi }(m,n(z_{m}),z_{m},c(z_{m}))}{\underbrace{\left(1-\tilde{\beta }(z_{m})\right)}}\;f_{\psi }(z_{m}\;|\;\Theta >0)dz_{m} \end{array}$$

(32) $$\begin{array}{ll} & \neq \int {\text{PP}}_{\varphi }(m,n(z_{m}),z_{m},c(z_{m}))\;f_{\varphi }(z_{m}\;|\;\Theta >0)dz_{m} \end{array}$$

(33) $$\begin{array}{ll} & ={\text{EP}}_{\varphi }(n(\cdot ),c(\cdot ))=1-\beta . \end{array}$$

### 3.2 “Reoptimization” approach

To additionally maintain the unconditional (expected) power during an unplanned sample size recalculation, $1-\tilde{\beta }(z_{m})$ needs to integrate to 1−β under the modified prior ψ≠φ. We propose to achieve this with a two‐step procedure. First, a modified version of the original optimization problem (20) to (22) is solved where the original prior φ is replaced with the updated prior ψ, and under the additional conditional type I error rate constraint dictated by the conditional error principle, that is

(34) $$\begin{array}{ll} \underset{n^{\prime \prime }(\cdot ),c^{\prime \prime }(\cdot )}{\text{argmin}}\; & E_{\psi }[n^{\prime \prime }(Z_{m})], \end{array}$$

(35) $$\begin{array}{ll} \text{subject to:}\; & {\text{Pr}}_{0}[Z_{n^{\prime \prime }(Z_{m})}>c^{\prime \prime }(Z_{m})]\leq \alpha , \end{array}$$

(36) $$\begin{array}{ll} & {\text{EP}}_{\psi }(n^{\prime \prime }(\cdot ),c^{\prime \prime }(\cdot ))\geq 1-\beta , \end{array}$$

(37) $$\begin{array}{ll} & \text{CP}(m,n^{\prime \prime }(z_{m}),z_{m},c^{\prime \prime }(z_{m}),0)\leq \text{CP}(m,n(z_{m}),z_{m},c(z_{m}),0). \end{array}$$

One could use $n^{\prime \prime }(z_{m})$ as the recalculated sample size and the corresponding critical value $c^{\prime \prime }(z_{m})$ directly. However, there may be cases where the conditional type I error rate constraint (37) is not binding thus leading to an unnecessarily low conditional error and a needlessly large sample size. Instead, as the second step, we propose to plug in $1-\tilde{\beta }(z_{m})={\text{PP}}_{\psi }(m,n^{\prime \prime }(z_{m}),z_{m},c^{\prime \prime }(z_{m}))$ in (27) and (28) and solve for $n^{\prime }$ and $c^{\prime }$. Since,

(38) $$\begin{array}{ll} 1-\beta & \leq {\text{EP}}_{\psi }(n^{\prime \prime }(\cdot ),c^{\prime \prime }(\cdot )) \end{array}$$

(39) $$\begin{array}{ll} & =\int \underset{1-\tilde{\beta }(z_{m})}{\underbrace{{\text{PP}}_{\psi }(m,n^{\prime \prime }(z_{m}),z_{m},c^{\prime \prime }(z_{m}))}}\;f_{\psi }(z_{m}\;|\;\Theta >0)dz_{m}\;, \end{array}$$

the *unconditional* expected power of the recalculation procedure maintains the original value of 1−β. Note that this only holds true if the choice of ψ is stochastically independent of $Z_{m}$, that is, if the change of the planning assumptions is not driven by the observed interim data.

### 3.3 Application example

In practice, group‐sequential designs are more common than optimal two‐stage designs. Since the efficiency gains of the latter over the simpler group‐sequential designs are often practically irrelevant this is a sensible approximation.^21^ Sample size calculations are still commonly based on point alternatives. This is equivalent to using a point prior on the alternative value. All previous considerations can thus be transferred to a setting with a fixed point alternative.

For this example, we consider the two‐stage, two‐arm group‐sequential design with binding early stopping for futility boundary at an observed effect of 0, which minimizes the expected sample size under the point alternative of a mean difference of $\theta_{1}=0.4$ under a one‐sided type I error rate constraint of α≤0.025 and an expected power of at least 0.8. Since the prior only has mass at $\theta_{1}=0.4$ this corresponds to a classical power constraint at $\theta_{1}=0.4$. We computed the optimal group‐sequential design by direct minimization over the stage‐one and two sample sizes and the stage‐two critical value using the R package nloptr.^22^

**FIGURE 4:**
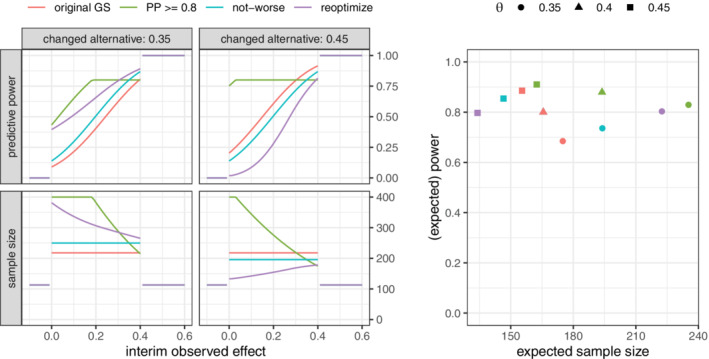
Left: Sample size (both arms) and predictive power for response‐independent adjustment of the alternative; predictive power is evaluated at the respective changed point alternative. Right: Unconditional (expected) power and expected sample size for the three scenarios when adapting to the respective true θ (for θ=0.4, the two consistent rules overlap with the original design)

We first consider a scenario where the alternative is modified during the interim analysis to either $\theta_{1}=0.35$ or $\theta_{1}=0.45$ *independently* of the observed interim data. The conditional and unconditional properties of three adaptation procedures together with those of the original design are shown in Figure 4. First, “PP ≥ 0.8” corresponds to a recalculation based on a fixed threshold for the predictive power of 0.8. As discussed earlier, this adaptation rule is not consistent in that the original design is not invariant under recalculation (see Section 2.2). Second, the “not‐worse” approach discussed in Section 3.1 instead matches the predictive power of the original design under the old prior. Third, the “reoptimize” approach discussed in Section 3.2 matches the predictive power of the design that is optimal under the new alternative. For all recalculations, the final adapted sample size was restricted to the range of 120 to 400. Clearly, “PP ≥ 0.8” is not only inconsistent but also the rule that leads to the most extreme adaptation of the sample size. The “not‐worse” approach leads to a level‐shift in the sample size with moderate increases or decrease of the stage‐two sample size. The “reoptimize” approach leads to more moderate increases in sample size as compared to the “PP ≥ 0.8” approach when the alternative is lowered and to substantial sample size reductions for an increased effect size. From an unconditional perspective, only the “reoptimize” approach maintains the target expected power of 0.8.

**FIGURE 5:**
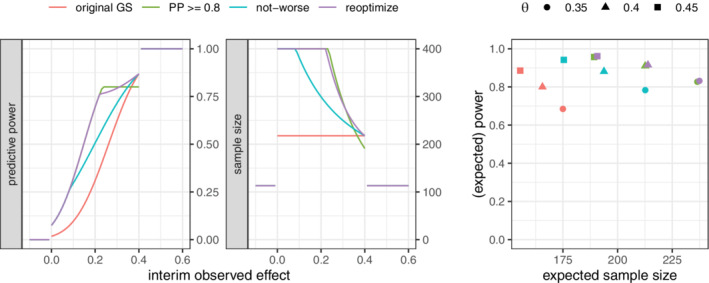
Left: Predictive power and sample size (both arms) for response‐adaptive modification of the alternative; predictive power is evaluated at the respective changed point alternative. Right: Unconditional (expected) power and expected sample size for three scenarios

A conceptual drawback of point priors is that they are invariant under formal Bayesian updating. Hence, the posterior distribution after observing the interim results is always the same point measure as the initial point prior This is merely a consequence of claiming perfect knowledge of the effect size during planning. In practice, investigators might still be inclined to modify the alternative at least heuristically during the interim analysis. We thus consider an alternative situation based on the same optimal group‐sequential design discussed above. Assume that the trial team wants to revise the strong assumption of a point prior on the alternative of a mean difference of 0.4 during the interim analysis using the observed interim data. For instance, this could be done heuristically by “updating” the alternative to the average of the observed effect and the originally assumed point alternative of 0.4. The resulting conditional and unconditional properties of the proposed recalculation rules are given in Figure 5. In this alternative scenario, the adapted sample size under all three rules is more variable and quickly reaches the maximal value of 400. The sample size is rarely reduced since the original group‐sequential design employs an aggressive early‐efficacy boundary of ≈0.4 on the scale of the observed treatment effect. Hence, the heuristically modified alternative almost never exceeds the original value of $\theta_{1}=0.4$. Since the adaptation is now response‐adaptive, the “reoptimize” approach does no longer maintain a power of 1−β.

These two example situations show that a consistent sample size adaptation can make sense as reaction to new trial‐external evidence. For the relevant case of reacting to new trial‐external evidence, it is possible to maintain the unconditional power properties of the original design. If, however, the change of alternative depends on the observed interim data, it is unclear how the unconditional power may be preserved.

## 4 DISCUSSION

Monitoring the power of the remainder of an ongoing trial constitutes an estimation problem since the conditional power is a function of the unknown treatment effect. This estimation problem can be formulated both conditional and unconditional on a positive treatment effect. In many practical situations, where the prior mass is concentrated on non‐null effects the consequences of conditioning are negligible but their importance increases with the vagueness of a the prior. The lack of conditioning on a positive treatment effect explains why observed conditional power performs rather poorly as an estimator of the unknown conditional power for a wide range of effect sizes. Assumed conditional power for $\theta_{1}>0$ does reflect this conditionality. It is, however, merely a special case of predictive power with a point prior on the effect size. In situations where the effect size is fairy well known, a point prior might constitute an acceptable, simpler approximation. In any other case, the Bayesian predictive power allows a priori information to be incorporated in a more fine‐grained way and guarantees optimal mean‐squared‐error performance. If an unconditional measure for the probability to reject the null hypothesis is sought, a conditional version of the probability of success (or assurance) can be derived in an analogue way.^11^

Great care should be taken when predictive power or another estimator of conditional power is used to modify an ongoing trial's sample size. Altering a simple starting design with a seemingly intuitive recalculation rule can lead to overall *less efficient* trials. Techniques originally intended for *unplanned* adaptations of trials should not be used for a *preplanned* sample size recalculation. Instead, an optimal two‐stage design should directly be derived for the objective criterion of interest. By definition, no trial‐internal event can then justify a sample size recalculation. Only trial‐external events, such as a change in the objective criterion, the emergence of new trial‐external evidence, or unforeseen deviations from the planned interim analysis time‐point may require a reassessment of the sample size of an optimal design.

A generic recalculation rule might lead to the paradoxical situation that an optimal starting design is always modified during the interim analysis—even if the planning assumptions remain unaltered throughout the trial. This is clearly ineffective and, consequently, an optimal starting design should be invariant under a sensible adaptation rule if the planning assumptions remain unchanged. This minimal consistency property is often not fulfilled when recalculating a design's sample size based on a fixed threshold for its minimal conditional power.

We proposed two consistent approaches to adjusting an ongoing optimal design to newly emerging trial‐external data. Both methods can be extended to deviations from the planned interim time‐point. The first method (“not‐worse”) applies the conditional error principle to the (average) type II error rate in a similar way to its use in controlling the maximal type I error rate. The method is easy to implement and consistent in the above defined way. However, it does not maintain the same unconditional power as the original design, not even under data independent modifications of the planning assumptions. The second method addresses this issue by adapting the sample size and critical value such that the predictive power matches the predictive power of a design that would have been optimal, had the new prior been known during the planning stage. As long as the prior is adapted data‐independently, this approach does indeed maintain the same unconditional power properties.

These considerations show how crucial the initial planning stage of a trial is. Adaptive methods should not be taken as an excuse to start with a sub‐optimal design and rely on a later sample size recalculation. Ideally, all uncertainty is quantified during the planning phase to the best possible extent and integrated in the initial design via a planning prior. A modification of the design is then only necessary and warranted when this planning prior changes. This does not mean that a complex design is always the best choice. If the variability of the final sample size and the operational burden of conducting interim analyses is penalized strong enough in the objective criterion, a simple one‐stage design might very well perform better than more complex alternatives. Generic optimal‐two stage designs require optimizing over function‐spaces to find the optimal sample size and critical value functions. Obtaining a stable solution might thus be hard in practice and group‐sequential designs are a viable approximation since optimizing the stage‐wise sample sizes and stopping boundaries only requires optimizing over a small set of real parameters. It is well‐known, that optimal group‐sequential designs approximate the performance of optimal two‐stage designs with variable sample sizes sufficiently well.^21^ Still, the same principle considerations apply: an optimal (group‐sequential) design only needs to be revised if either the objective function or the underlying planning assumptions, that is, the prior on the effect size, changes.

A limitation of the proposed recalculation methods is the fact that they rely on the conditional error principle for strict type I‐error rate control. The conditional error principle can be difficult to extend to cases with nuisance parameters.^23^ In practice, a simple plug‐in approach similar to the approach in blinded sample size reassessment might be viable but has not yet been investigated more thoroughly. Alternatively, any other form of pre‐specifying a combination function for the stage‐wise *P*‐values might be used to control the type I‐error rate. This combination function should then also be optimized over during the planning stage to avoid inconsistencies. In particular, the optimal combination function of a two stage‐design minimizing expected sample size can be approximated with an inverse normal combination test.^9^

## Data Availability

The source code required to reproduce all results presented in this article is available at github.com and zenodo.org.
